# Draft genomes of 12 *Ralstonia mannitolilytica* isolates recovered from hospitalized patients and contaminated cosmetic water sprays

**DOI:** 10.1128/mra.00300-25

**Published:** 2025-06-20

**Authors:** Trestan Pillonel, Dominique Blanc, Damien Jacot, Florian Mauffrey, Estelle Ben Salah-Moulin, Laurence Senn, Gilbert Greub, Claire Bertelli

**Affiliations:** 1 Institute of Microbiology, Lausanne University Hospital and University of Lausanne, Lausanne, Switzerland; 2 Infection Prevention and Control Unit, Infectious Diseases Service, Lausanne University Hospital and University of Lausanne, Lausanne, Switzerland; Loyola University Chicago, Chicago, Illinois, USA

**Keywords:** genomics, microbial source tracking, epidemiology

## Abstract

*Ralstonia mannitolilytica,* an opportunistic pathogen commonly present in water, was identified in three hospitalized patients over a short period of time. The comparison of draft genomes of 12 isolates from patients and water sprays showed fewer than 10 single-nucleotide polymorphisms, indicating that water sprays were the contamination source.

## ANNOUNCEMENT

*Ralstonia mannitolilytica* is an opportunistic pathogen commonly present in various water supplies (1). *Ralstonia* spp. have previously been implicated in hospital outbreaks and are often associated with contaminated liquids (1). Here, we report the draft genome assemblies of 12 *R*. *mannitolilytica* isolates: three obtained from three different patients and nine from three distinct batches of cosmetic water sprays used for patient comfort at the Lausanne University Hospital, Switzerland (geographical coordinates 46.524866934584246, 6.642684751705649). This work was conducted under the local ethics committee (CER-VD) agreement on quality studies. Written informed consent from the participants’ legal guardian/next of kin was not required to participate in this study in accordance with the national legislation and the institutional requirements.

Patient and environmental samples were cultured on Columbia blood agar, incubated at 35°C in a natural atmosphere for 48 hours. Identification of isolated colonies was performed with MALDI-ToF (Bruker, Germany). After two subcultures from single colonies to ensure isolate purity, genomic DNA was extracted with a QIAcube Connect MDX using the QIAamp DNA Mini Kit (Qiagen, ref. 51306). DNA quantification on a Qubit (Thermo Fisher Scientific, USA) indicated an average concentration of 25.6 ng/uL (range 12.4–73.8 ng/uL). Libraries were prepared using the DNA Prep kit (Illumina, USA). Sequencing was performed on a MiSeq platform (Illumina, USA), generating 151 or 251 bp paired-end reads (Table 1). Reads were assembled using an in-house Snakemake workflow (https://github.com/metagenlab/diag_pipelines, version 2.8.3). Briefly, read quality was assessed with FastQC version 0.11.8 (http://www.bioinformatics.babraham.ac.uk/projects/fastqc/). Reads were trimmed with Trimmomatic version 0.32 (SLIDINGWINDOW:5:20 LEADING:28 TRAILING:28 MINLEN:50) (2) and *de novo* assembled with SPAdes 3.15.4 (-k 21,33,55,77,99,111,127) (3). Contigs smaller than 500 bp and with a sequencing depth lower than 5% of the median depth of the full assembly were filtered out. The completeness and contamination of individual assemblies were assessed with checkM version 1.0.20 based on the presence of 596 *Burkholderiaceae* marker genes (4). Genome assemblies were annotated with Bakta version 1.9.3 (--compliant mode) (5). Antimicrobial resistance genes were identified with RGI version 5.2.1 (database version 3.2.2) (6). Trimmed reads were mapped on the *de novo* assembly of the strain GEN20273 with bwa mem (7). Single-nucleotide polymorphisms were identified with GatK HaplotypeCaller (--sample-ploidy 1 -ERC BP_RESOLUTION) (8). Variants were filtered with bcftools filter (9), keeping only single-nucleotide polymorphisms (SNPs) supported by 75% of the reads and a sequencing depth of at least 10×. Detailed commands of each step of the analysis can be retrieved from the code of the Snakemake workflow.

**TABLE 1:** List of bacterial isolates with references to publicly available data and genome assembly characteristics

Isolate identifier	Isolation source	Collection date	SRA accession	Assembly accession	Read pairs (millions)	Read length (bp)	Median depth	# contigs (≥500 bp)	Total length	Largest contig	GC (%)	N50	CheckM completeness (%)	CheckM contamination (%)
GEN20269	Blood sample	2024-06-04	ERS22556679	GCA_965118695.1	0.8	251	37	15	4,962,648	1,430,648	65.75	778,894	99.94	1.07
GEN20273	Bronchial fluid sample	2024-05-28	ERS22556680	GCA_965118585.1	1	251	56	14	4,963,342	1,430,648	65.75	1,301,517	99.94	1.07
GEN20286	Anal smear sample	2024-06-18	ERS22556681	GCA_965118565.1	3.2	151	134	13	4,963,401	1,722,884	65.75	1,430,649	99.94	1.07
GEN20613	Water spray 1	2024-07-17	ERS22556682	GCA_965118805.1	1.1	251	78	11	4,962,910	1,430,649	65.75	1,345,105	99.94	1.07
GEN20614	Water spray 1	2024-07-17	ERS22556683	GCA_965118955.1	1.1	251	77	14	4,962,850	1,121,388	65.75	778,894	99.94	1.07
GEN20615	Water spray 1	2024-07-17	ERS22556684	GCA_965118935.1	2	251	118	11	4,962,916	1,679,297	65.75	1,430,648	99.94	1.07
GEN20616	Water spray 2	2024-07-17	ERS22556685	GCA_965118355.1	1.7	251	100	11	4,962,917	1,679,297	65.75	1,430,648	99.94	1.07
GEN20617	Water spray 2	2024-07-17	ERS22556686	GCA_965118615.1	1.1	251	73	13	4,962,870	1,430,648	65.75	1,182,931	99.94	1.07
GEN20618	Water spray 2	2024-07-17	ERS22556687	GCA_965118705.1	1.1	251	72	11	4,962,828	1,430,648	65.75	1,226,518	99.94	1.07
GEN20619	Water spray 3	2024-07-22	ERS22556688	GCA_965118515.1	1.2	251	83	11	4,962,857	1,639,390	65.75	1,430,648	99.94	1.07
GEN20620	Water spray 3	2024-07-22	ERS22556689	GCA_965118345.1	1	251	66	10	4,962,900	1,722,884	65.75	1,430,648	99.94	1.07
GEN20621	Water spray 3	2024-07-22	ERS22556690	GCA_965118775.1	1.1	251	77	12	4,962,882	1,430,648	65.75	1,301,506	99.94	1.07

**Fig 1:**
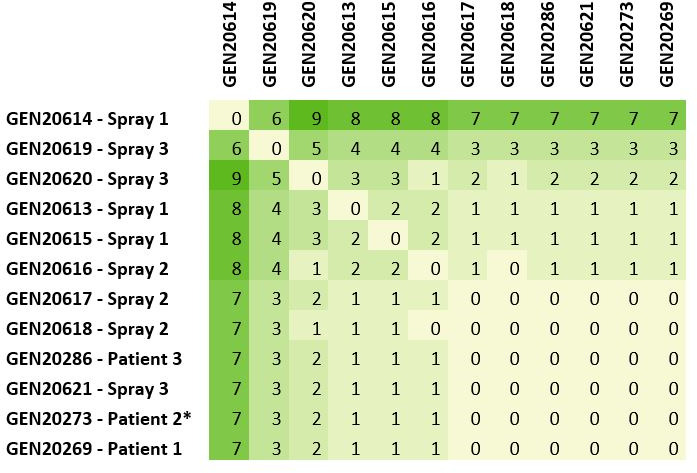
Pairwise SNP distance. The * indicates the genome used as a reference to perform the comparison between isolates from the patient and the water sprays.

The genome size of the 12 draft assemblies ranged from 4,962,648 to 4,963,401 bp and assemblies were split into 10 to 15 contigs, with a median sequencing depth of 37× to 134× (Table 1). All assemblies were predicted to be highly complete and not contaminated. All isolates carry OXA-443 (OXA-22-like) and OXA-444 (OXA-60-like) β-lactamases, which are known to be chromosomally encoded in this species (10). SNP-based comparisons showed that all *R. mannitolilytica* isolates are genetically highly similar (Fig. 1). Six of the 12 isolates, including the three patient isolates and three water spray isolates, are strictly identical, suggesting that the water sprayers were the source of the contamination. The other six isolates from water showed between 1 and 7 polymorphisms with the patient isolates, and between 0 and 9 SNPs among each other, demonstrating the existence of some genetic diversity within the *R. mannitolilytica* population present in the water sprays.

## Data Availability

The standardized isolate descriptions and accession numbers are presented in Table 1; the genomic data are publicly available in DDBJ/ENA/GenBank under BioProject no. PRJEB82839. The versions described are the first versions.
